# The AhR regulates IFN-induced immune checkpoints in lung cancer cells through HNRNPH1, an RNA-binding protein, and *INCR1*, a novel long non-coding RNA

**DOI:** 10.1016/j.jbc.2025.110316

**Published:** 2025-05-29

**Authors:** Brian Lara, Megan Snyder, Jocelyn Fimbres, Eric Yang, Gang Song, Vinay Kumar Duggineni, Zhongyan Wang, David H. Sherr

**Affiliations:** 1 Department of Environmental Health, Boston University School of Public Health, Boston, Massachusetts, USA; 2 Triveni Bio, Watertown, Massachusetts, USA

**Keywords:** aryl hydrocarbon receptor, checkpoint control, gamma interferon, immunotoxicology, long noncoding RNA, lung cancer, polycyclic aromatic hydrocarbons

## Abstract

Although immune checkpoint inhibitors show great promise, not all patients respond, and many do not achieve durable responses. Consequently, further investigations into potentially targetable molecules that regulate immune checkpoints are warranted. Previous studies in several cancers demonstrated that interferons produced by tumor-infiltrating leukocytes regulate immunosuppressive PD-L1, PD-L2, and IDO1 through JAK/STAT signaling. Here, we investigated a novel role for an immunosuppressive environmental chemical receptor, previously implicated in smoking-related cancers, in IFN signaling in human lung adenocarcinoma (LUAD) cells. Deletion of the aryl hydrocarbon receptor (AhR) from A549 LUAD cells significantly decreased baseline JAK2, STAT1, STAT3, IRF1 (a JAK/STAT target), PD-L1, PD-L2, and IDO1 expression. IFNγ and IFNα increased the expression of JAK/STAT and immune checkpoint genes and proteins, but these increases were significantly diminished or absent in AhR-knockout cells. The AhR similarly controls IFN-induced, JAK/STAT-driven increases in multiple MHC class I- and class II-related genes. AhR control of type I and type II interferon signaling is mediated through up-regulation of an lncRNA, the IFN-stimulated non-coding RNA 1 (*INCR1*), and through repression of an RNA-binding protein, heterogeneous nuclear ribonucleoprotein H1 (HNRNPH1), which sequesters JAK/STAT-related and immune checkpoint gene transcripts. The data suggest that the AhR is a key mediator of tumor immunosuppression through regulation of IFN-induced INCR1 and JAK/STAT signaling and, thereby, expression of immune checkpoints. However, that immunosuppression may be tempered by AhR control of MHC expression. Given the multiple roles of JAK/STAT signaling in the immune system, the results also suggest multiple levels at which the AhR may affect tumor immunity.

Non-small cell lung cancer (NSCLC) is the third most commonly diagnosed human cancer (1) with lung adenocarcinomas (LUADs) making up the majority of NSCLC diagnoses (2). NSCLC causes more than 350 deaths in the United States each day, the highest number of deaths for any cancer type (3). In recent years, immune checkpoint inhibitors (ICIs), particularly inhibitors of the PD1-PD-L1 axis, have improved patient survival (4, 5, 6). However, not all patients are eligible for ICI treatment, not all treated patients respond, and not all responses are durable (4, 7, 8). It is important, therefore, to further map out molecular signaling pathways that regulate the expression of immune checkpoints (or other immunosuppressive signals), and to identify, along those pathways, new targets for immunoenhancement.

Previous studies implicate the JAK/STAT pathway in the control of PD-L1 expression by lung cancer cells (9, 10, 11). Paradoxically, IFNγ, a T and NK cell cytokine generally associated with immune enhancement and present in the tumor microenvironment (TME) (11, 12, 13), activates the JAK/STAT pathway, thereby increasing PD-L1 expression and driving immunosuppression. PD-L2, a somewhat less well-studied immune checkpoint, is similarly induced by IFNγ (14) through a super-enhancer that drives synchronous transcription of both *CD274* (*PD-L1*) and *PDCD1LG2* (*PD-L2*) (15). IFNγ and the JAK/STAT pathway also drive production of indoleamine-2,3-dioxygenase (IDO1), a proximal and rate-limiting enzyme in the kynurenine pathway of tryptophan metabolism (16). IDO1 and tryptophan metabolites, including kynurenine itself, are immunosuppressive in a variety of cancers (17). Hence, IFNγ regulates at least three critical immunosuppressive factors, PD-L1, PD-L2, and IDO1.

Recently, novel regulators of the JAK/STAT pathway, PD-L1 and IDO expression, have been identified (18). Mineo *et al.* identified an IFNγ-induced long non-coding RNA (lncRNA) referred to as the IFNγ-stimulated non-coding RNA 1 (*INCR1*) (18). Furthermore, *INCR1* knockdown significantly reduced PD-L1 expression and increased cytotoxic T cell function in the context of glioblastoma (19). This lncRNA is encoded across a >150 kb span of the PD-L1/PD-L2 locus and is transcribed from the antisense strand in the presence of IFNγ (18). *INCR1* binds to and blocks HNRNPH1, a nuclear RNA-binding protein that sequesters *PD-L1, JAK2, IDO1*, and other gene transcripts, thereby preventing their translation into protein. Therefore, molecules that control *INCR1* and HNRNPH1 expression and/or function are likely to contribute significantly to the regulation of tumor immunity.

Here, we investigated the likelihood that the aryl hydrocarbon receptor (AhR), an environmental carcinogen-activated receptor/transcription factor linked to immunosuppression (20, 21), is involved in JAK/STAT signaling in general and *INCR1* and HNRNPH1 expression in specific. Historically, the AhR was studied for its ability to bind to and be activated by whole classes of environmental chemicals, including dioxins and polycyclic aromatic hydrocarbons (PAH), the latter frequently associated with lung carcinogenesis and immunosuppression. More recent studies have shown the AhR’s role in skewing immune cells, including T cells (22, 23, 24, 25), macrophages (26), and dendritic cells (27, 28), toward immunosuppressive phenotypes. Several studies demonstrated that the AhR is transcriptionally active in malignant cells in the absence of environmental ligands through tumor production of endogenous AhR ligands, at least some of which are likely to be derived from the kynurenine (Kyn) pathway of tryptophan metabolism (29, 30, 31). Production of these tryptophan metabolites is dependent, to a large extent, on proximal tryptophan dioxygenases, including IDO1, which itself is associated with cancer immunosuppression and has been targeted for cancer immunotherapy (32). Like PD-L1 and PD-L2, IDO1 is upregulated by Type I and Type II IFNs through JAK/STAT signaling (33, 34, 35).

To assess the possible involvement of the AhR in the IFNγ→JAK/STAT→immune checkpoint pathway, and to determine the contribution, if any, of *INCR1* and HNRNPH1 to PD-L1, PD-L2, and IDO expression in LUAD, the AhR was deleted from human (A549) and mouse (CMT167) LUAD cells, and the effect of IFNγ treatment on the JAK/STAT pathway was determined. The study was also extended to a type I interferon (IFNα) and to other immune-related target genes of the JAK/STAT pathway, *i.e.*, MHC Class I- and MHC Class II-related genes. The results suggest a central and complex role for the AhR in controlling tumor immunity.

## Results

### RNA-seq studies indicate that the AhR is a key regulator of IFN**γ**-induced JAK/STAT signaling in a human LUAD model

**Figure 1:**
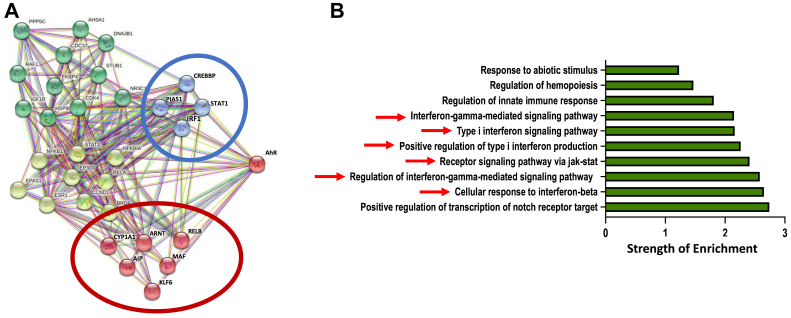
**An AhR-directed STRING interactions network predicts crosstalk between the AhR and components of interferon signaling.***A*, “*Human-AhR”* was used as the input term to build a STRING protein-protein and gene-protein network using K-means clustering (requesting n = 4 clusters) to determine predicted AhR protein-protein or protein-gene interactions. These interactions were generated from sequenced genomes that define common genomic neighborhoods, co-expression of genes, and gene fusion occurrences. *B*, Gene Ontology (GO) enrichment analysis of the AhR-interferon signaling network circled in *blue* in Figure 1*A*. The top ten most highly enriched pathways are shown.

We previously noted that the AhR is highly expressed in primary LUAD cells (36, 37). Furthermore, its nuclear localization (36, 37) and its contribution to oncogene PLK1 (Polo-like kinase1)-mediated epithelial-to-mesenchymal transition in LUAD (38) suggest that the AhR is chronically active in these tumors. To characterize the signaling pathways impacted by the AhR, we built a gene and protein interaction network using the Search Tool for the Retrieval of Interacting Genes/Proteins (STRING) database (39). The STRING database takes established gene and protein interaction pathways to compute a predicted interaction network (39). Using k-means clustering and “*human-AhR”* as an input term, we identified four nodes predicted to directly interact with the human AhR (Fig. 1*A*). One node (red circle) included canonical components of AhR signaling, such as the quintessential AhR target gene encoding cytochrome P4501A1 (*CYP1A1*), the AhR nuclear translocator (ARNT), *MAF*, which we demonstrated to be a direct gene AhR target in Tr1 regulatory T cells (24), and three AhR protein-binding partners (AIP, KLF6, RelB), each of which regulates AhR activity in various contexts (40, 41, 42). A second directly interacting node (blue circle) included STAT1, interferon regulatory factor 1 (IRF1, which is transcriptionally induced by STAT1 and further drives STAT1 activity (43)), the STAT co-activator CREBB binding protein (CREBBP), and the protein inhibitor of activated STAT 1 (PIAS1). A Gene Ontology (GO) enrichment analysis of biological function (44) using STAT1, IRF1, CREBB, and PIAS1 as input terms further implicated a significant enrichment for Type I and Type II IFN-mediated signaling *via* JAK/STAT signaling as an underlying AhR-linked biological process (Fig. 1*B*, red arrows).

**Figure 2:**
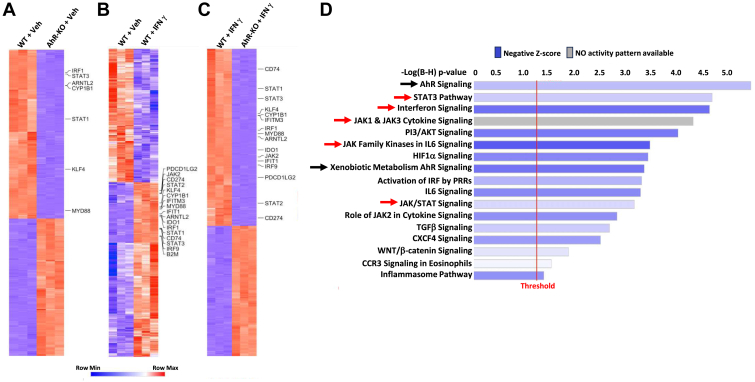
**Genome-wide analysis of baseline and IFNγ-induced, AhR-regulated genes in human LUAD cells.** RNA was extracted from three biological replicates of A549^WT^ control or three biological replicates of A549^AhR-KO^ cells treated for 24 h with vehicle (0.1% PBS) or 100 ng/ml IFNγ, reverse transcribed, and cDNA sequenced using the Illumina NextSeq 2000 platform. Resulting counts were defined as the number of read pairs aligning uniquely to the genome in proper pairs and then assigned to a single Ensembl Gene locus for each gene transcript. Heatmaps were generated using the 5000 most significantly differentially expressed genes (sorted by lowest *p* values). *A*, heatmap of differentially expressed genes (DEGs) from vehicle-treated A549^AhR-KO^ cells as compared with vehicle-treated A549^WT^ cells (*i.e.*, baseline AhR-regulated genes) (*p* < 10^−6^). *B*, heatmap of DEGs from IFNγ-treated A549^WT^ cells as compared with vehicle-treated A549^WT^ cells (*i.e.*, IFNγ-inducible genes) (*p* < 0.005). *C*, heatmap of DEGs from IFNγ-treated A549^AhR-KO^ cells as compared with IFNγ-treated A549^WT^ (*i.e.*, IFNγ-inducible, AhR-regulated genes) (*p* < 10^−29^). *D*, ingenuity pathway analysis (IPA) of DEGs (FDR < 0.05) from IFNγ-treated A549^AhR-KO^ cells as compared with IFNγ-treated A549^WT^ cells (*i.e.*, IFNγ-inducible, AhR-regulated genes). *Black* arrows call out AhR signaling pathways. *Red* arrows call out interferon and JAK/STAT pathways.

To determine whether the AhR is causally linked to the JAK/STAT or other IFNγ-induced signaling pathways in a LUAD model, we generated AhR knockout human A549 (A549^AhR-KO^) LUAD cells by CRISPR/Cas9 gene editing and treated them, along with wild-type control (A549^WT^) cells, with vehicle (0.1% PBS) or 100 ng/ml IFNγ. Twenty-four hours later, mRNA was isolated and sequenced to identify differentially expressed genes (DEGs). Heatmaps were generated showing DEGs in comparisons of: (A) vehicle-treated A549^WT^ cells *vs* PBS-treated A549^AhR-KO^ cells (*i.e.*, baseline AhR-controlled genes), (B) PBS-treated A549^WT^ cells *vs* IFNγ-treated A549^WT^ cells (*i.e.*, IFNγ-induced genes), and (C) IFNγ-treated A549^WT^ cells *vs* IFNγ-treated A549^AhR-KO^ cells (*i.e.*, AhR controlled IFNγ-induced genes) (Fig. 2, *A*–*C*, Table 1, Columns A,B,C).

We noted significant (adjusted *p* < 10^−9^) decreases in baseline levels of four JAK/STAT pathway genes (*STAT1*, *STAT3*, *IRF1*, and *JAK2*) after AhR deletion (Table 1, Column A). Similarly, levels of two immune checkpoint genes, *CD274(PD-L1)* and *PDCD1LG2 (PD-L2)*, and several MHC-related genes including *CD74* (MHC II invariant chain), *HLA-A*, *B2M* (β2 microglobulin), and *CIITA* (MHC class II transactivator), were significantly lower in A549^AhR-KO^ cells (Table 1, Column A). All of these genes, in addition to several other JAK/STAT pathway genes (*STAT1, STAT2, IRF9, IFITM3, and IFIT1)* and immune-related (*IDO1*, *MYD88*) genes, were induced on addition of IFNγ to A549^WT^ cells (Table 1, Column B). Notably, expression of all of the IFNγ-inducible JAK/STAT-related and IFNγ-inducible immune-related genes listed above was significantly diminished (*p* < 10^−12^) in A549^AhR-KO^ cells relative to controls (Table 1, Column C).

**Table 1:** JAK/STAT- and immune-related genes downregulated in AhR-knockout LUAD cells in the absence or presence of IFNγ

			A		B		C	
			(Wt PBS) vs (AhR-KO + PBS) (baseline genes down-regulated in AhR-KO cells)		(Wt PBS) vs (WT + IFNγ) (IFNg-induced genes)		(WT + IFNγ) vs (AhR-KO + IFNγ) (IFNγ-induced genes down-regulated in AhR-KO cells)	
			Avg.Log_2_-fold change	Adjusted *p* value	Avg.Log_2_-fold change	Adjusted *p* value	Avg.Log_2_-fold change	Adjusted *p* value
JAK/STAT/IRF Signaling	*STAT1*	JAK/STAT signaling	−1.9	1.2 × 10^−11^	3.4	1.4 × 10^−8^	−3.0	5.6 × 10^−15^
	*STAT2*	JAK/STAT signaling	0.2	0.018	3.3	4.3 × 10^−9^	−2.2	1.0 × 10^−14^
	*STAT3*	JAK/STAT signaling	−1.9	1.1 × 10^−10^	1.3	1.6 × 10^−6^	−2.1	6.8 × 10^−14^
	*JAK2*	JAK/STAT signaling	−0.8	2.0 × 10^−5^	2.7	1.6 × 10^−8^	−3.3	6.6 × 10^−14^
	*IRF1*	JAK/STAT targeted gene	−1.9	1.7 × 10^−9^	5.5	3.4 × 10^−10^	−3.8	5.8 × 10^−16^
	*IRF9*	JAK/STAT targeted gene	−0.3	NS	3.2	3.0 × 10^−8^	−2.3	3.6 × 10^−14^
	*IFITM3*	JAK/STAT targeted gene	0.7	0.001	4.7	7.0 × 10^−9^	−2.4	5.7 × 10^−13^
	*IFIT1*	JAK/STAT targeted gene	0.6	0.001	3.8	1.1 × 10^−8^	−2.7	2.2 × 10^−13^
Immune Function	*CD274*	PD-L1, Immunosuppression	−2.5	4.0 × 10^−5^	5.0	9.1 × 10^−8^	−5.1	2.4 × 10^−12^
	*PDCD1LG2*	PD-L2, Immunosuppression	−4.4	0.0004	5.5	1.9 × 10^−6^	−9.3	4.1 × 10^−8^
	*IDO1*	Immunosuppresssive enzyme in the kynurenine pathway, generates AhR ligands	0.0	NS	17.5	1.9 × 10^−7^	−11.1	5.7 × 10^−15^
	*MYD88*	Innate immune signal transduction adaptor	−6.3	6.1 × 10^−11^	1.3	7.2 × 10^−7^	−6.9	1.6 × 10^−12^
	*KLF4*	AhR target in suppressive macrophages	−1.5	1.0 × 10^−7^	1.5	2.81.6 × 10^−6^	−2.7	3.6 × 10^−12^
MHC-related	*HLA-A*	MHC-Class I	−5.8	3.2 × 10^−8^	2.5	4.6. × 10^−7^	−8.0	3.7 × 10^−11^
	*B2M*	MHC-Class I component	−0.6	2.1 × 10^−5^	2.9	2.3 × 10^−8^	−2.7	7.1 × 10^−11^
	*CTIIA*	MHC-Class II transactivator	−0.4	NS	6.6	3.5 × 10^−8^	−6.2	5.8 × 10^−13^
	*CD74*	MHC II invariant chain required for antigen presentation	0.4	NS	11.0	3.8 × 10^−8^	−9.7	1.4 × 10^−13^

A549^WT^ or A549 ^AhR-KO^ cells were treated with vehicle or 100 ng/ml IFNγ. RNA was harvested 24 h later and subjected to bulk RNA sequencing. Column A) Comparison of RNA from vehicle-treated A549 ^AhR-KO^*vs* vehicle-treated A549^WT^ cells (baseline AhR-regulated genes). Column B) Comparison of RNA from vehicle-*vs* IFNγ-treated A549^WT^ cells (IFNγ-inducible genes). Column C) Comparison of RNA from IFNγ-treated A549^AhR-KO^*vs* IFNγ-treated A549^WT^ cells (AhR regulated, IFNγ-induced genes).

Ingenuity pathway analysis (IPA) of all significantly downregulated genes in IFNγ-treated A549^AhR-KO^ cells, as compared with IFNγ-treated A549^WT^ cells (FDR < 0.05), indicated that, as expected, the AhR signaling pathway was the most significantly downregulated pathway (Fig. 2*D*, black arrows). IPA also implicated the IFN signaling and the JAK/STAT/IRF pathway as being among the most highly downregulated pathways in IFNγ-treated A549^AhR-KO^ cells relative to IFNγ-treated controls (Fig. 2*D*, red arrows). Also of note were two other signaling pathways associated with tumor aggression, the TGFβ and WNT/β-catenin pathways, both of which are dysregulated in LUAD (45, 46) and are induced in normal lung epithelial cells exposed to environmental AhR ligands (*i.e.*, PAH) (47).

These RNA-seq data suggest an important role for the AhR in controlling multiple immune-related outcomes, most particularly the IFNγ-driven JAK/STAT/IRF pathway known to regulate IDO, PD-L1, and PD-L2 expression (9, 11, 48).

### The AhR regulates baseline and type II interferon (IFN**γ**)-induced levels of JAK/STAT and immune checkpoint-related genes

**Figure 3:**
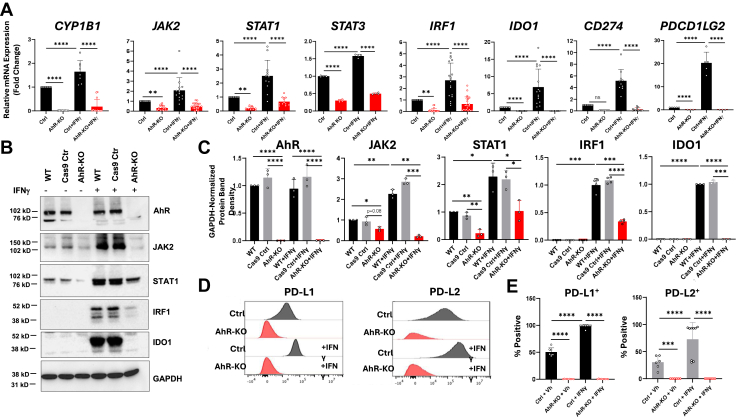
**The AhR regulates baseline and IFNγ-induced levels of JAK2, STAT1, STAT3, IRF1, IDO1, PD-L1, and PD-L2.** Control or A549^AhR-KO^ cells were treated for 24 h with vehicle (0.1% PBS) or 100 ng/ml IFNγ. *A*, RT-qPCR quantification of a prototypic AhR-target gene (*CYP1B1*), JAK/STAT-related genes (*JAK2*, *STAT1*, *STAT3*, *IRF1*), and immune checkpoint genes (*IDO1*, *CD274*, *PDCD1LG2*). (There were no statistical differences here or elsewhere between gene levels in A549^WT^ and A549^Cas9^ cells. Therefore, results from those two control lines were pooled and referred to here and elsewhere as “Ctrl”). Data from two-six experiments, each in duplicate or triplicate, are presented as mean fold-change of *18s RNA*-normalized mRNA expression + SD. ∗*p* < 0.05, ∗∗*p* < 0.01, ∗∗∗∗*p* < 0.001, Two-way ANOVA with *post hoc* Tukey test. *B*, representative (n = 3) western immunoblot for AhR, JAK2, STAT1, IRF1, IDO1, and GAPDH protein levels in vehicle or IFNγ-treated A549^WT^, A549^Cas9^, or A549^AhR-KO^ cells. *C*, data averaged from three independent western blotting experiments are presented as mean fold-change of GAPDH-normalized band densities + SD. ∗*p* < 0.05, ∗∗*p* < 0.01, ∗∗∗*p* < 0.001, ∗∗∗∗*p* < 0.0001 (Student’s *t* test, unpaired, two-tailed). *D*, a representative flow cytometry plot of A549^Ctrl^ or A549^AhR-KO^ cells treated with vehicle or IFNγ for 24 h and stained for PD-L1 or PD-L2 expression. *E*, data averaged from two to three experiments, each in duplicate or triplicate, in which cells were treated as in (*D*) are presented as percent PD-L1^+^ or PD-L2^+^ cells + SD. ∗*p* < 0.05, ∗∗*p* < 0.01, ∗∗∗*p* < 0.001, ∗∗∗∗*p* < 0.0001 (two-way ANOVA with *post hoc* Tukey test).

To confirm and extend the RNA-seq data, A549^AhR-KO^ cells, control A549 cells transduced with Cas9 without guide RNA (A549^Cas9^), or control wild-type (A549^WT^) cells were treated with PBS (0.1%) or 100 ng/ml IFNγ for 24 h and the *18s* RNA-normalized levels of *CYP1B1*, *JAK2*, *STAT1*, *STAT3*, *IRF1, IDO1*, *CD274*, and *PDCD1LG2* quantified by RT-qPCR. (No differences were seen in results with A549^Cas9^ and A549^WT^ cells. Therefore, data from the two lines were pooled and generically referred to as “Ctrl” cells here and elsewhere. Baseline levels of all eight genes were significantly lower in PBS-treated A549^AhR-KO^ as compared with A549^ctrl^ cells (Fig. 3*A*, first 2 bars in each graph), suggesting that constitutively active (endogenous ligand-driven) AhR regulates baseline JAK/STAT signaling and the resulting expression of immunosuppressive *IDO1*, *CD274,* and *PDCD1LG2*. IFNγ significantly increased expression of all seven JAK/STAT-related genes (Fig. 3*A*, third bars). Surprisingly, IFNγ also increased *CYP1B1* levels, a result that likely reflects IFNγ induction of IDO1, a proximal enzyme in the production of tryptophan-derived AhR ligands (49). IFNγ-driven increases in all eight genes were muted or completely absent in IFNγ-A549^AhR-KO^ cells (Fig. 3*A*, fourth bar in each graph).

As expected, AhR protein was seen by Western blotting in control A549^WT^ and A549^Cas9^ cells but not in A549^AhR-KO^ cells (Fig. 3, *B* and *C*). Baseline levels of JAK2 and STAT1 protein were lower in A549^AhR-KO^ as compared with control A549^Cas9^ and A549^WT^ cells (Fig. 3, *B* and *C*). While baseline *IRF1* and *IDO1* mRNAs were detectable by RT-qPCR (Fig. 3*A*), baseline levels of IRF1 and IDO1 protein were not detected by Western blotting. However, IFNγ significantly increased the levels of IRF1 and IDO1 as well as JAK2 and STAT1 protein in control cells (Fig. 3, *B* and *C*). Notably, IFNγ induction of all four proteins was significantly (JAK2, STAT1, IRF1) or completely (IDO1) reduced in IFNγ-treated A549^AhR-KO^ cells (Fig. 3, *B* and *C*). The baseline and IFNγ-induced percentages of PD-L1^+^ and PD-L2^+^ cells, as measured by flow cytometry, were significantly lower in PBS- and IFNγ-treated A549^AhR-KO^ cells as compared with their respective IFNγ-treated control cells (Fig. 3, *D* and *E*). These data indicate that the AhR generally controls the baseline (endogenous AhR ligand-driven) and IFNγ-induced levels of JAK/STAT genes/proteins and, likely through that pathway, regulates the immune checkpoints IDO1, PD-L1, and PD-L2.

### The AhR regulates baseline and type I interferon (IFN**α**)-induced levels of *STAT1*, *STAT2*, *IRF9*, *Ido1*, *CD274*, and *PDCD1LG2*

**Figure 4:**
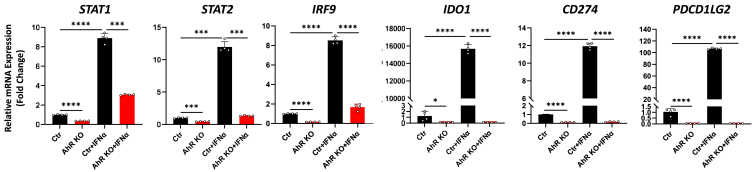
**The AhR regulates IFNα-induced *STAT1*, *STAT*2, *IRF9*, *IDO1*, *CD274*, and *PDCDLG2* expression levels.** A549^Ctrl^ or A549^AhR-KO^ cells were treated for 24 h with vehicle (0.1% PBS) or 100 ng/ml IFNα, RNA extracted, and JAK/STAT- and immune checkpoint-related genes quantified by RT-qPCR. Data from two experiments, each in duplicate, are presented as mean fold-change of *18s* RNA-normalized mRNA levels + SD. ∗*p* < 0.05, ∗∗∗*p* < 0.001, ∗∗∗∗*p* < 0.0001 (Student’s unpaired, two tailed *t* test).

Type I IFNs are also known to induce IDO, PD-L1, and PD-L2 through a canonical JAK/STAT signaling, albeit through a modestly different set of JAK/STAT components (*e.g.*, STAT1, STAT2, IRF9) than Type II IFN (50). To assess if the AhR regulates JAK/STAT genes in the IFNα signaling pathway, A549^Ctrl^ and A549^AhR-KO^ cells were treated with vehicle or 100 ng/ml IFNα for 24 h, and expression of *STAT1*, *STAT2*, *IRF9*, *IDO1*, *CD274*, and *PDCD1LG2* was quantified by RT-qPCR. As in the previous experiments, baseline levels of all these genes were significantly decreased in A549^AhR-KO^ as compared with control cells (Fig. 4, first 2 bars in each graph). IFNα treatment of A549^Ctr^ cells significantly induced all of these genes in an AhR-dependent fashion (Fig. 4, last 2 bars in each graph). These results extend those obtained with IFNγ and indicate that type I and type II IFN-induced outcomes, including regulation of critical immune checkpoints, are AhR-regulated.

### The AhR regulates human and murine MHC class I- and MHC class II-related molecules

**Figure 5:**
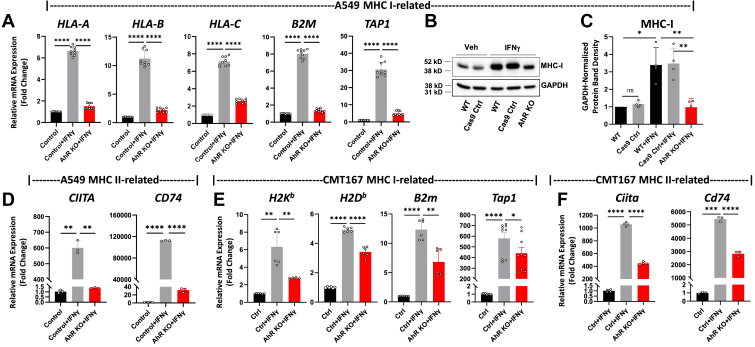
**The AhR regulates IFNγ-inducible MHC and MHC-related genes in human and mouse LUAD cells.***A*, A549^Ctrl^ and A549^AhR-KO^ cells were treated with vehicle or 100 ng/ml IFNγ for 24 h and MHC class I and related genes quantified by RT-qPCR. Data from three experiments, each in triplicate, are expressed as mean fold-change of *18s RNA*-normalized mRNA levels + SD. *B*, representative Western blot (n = 3), using GAPDH- and pan HLA-specific antibodies, of protein from A549^wt^, A549^Cas9^, or A549^AhR-KO^ cells treated for 24 h with vehicle or IFNγ. (An irrelevant AhR band at 105 kd was removed for clarity). *C*, data averaged from three independent western blotting experiments, one in duplicate, performed as in (*B*) are presented as mean fold-change of GAPDH-normalized band densities + SD. *D*, A549^Ctrl^ and A549^AhR-KO^ cells were treated with vehicle or IFNγ and MHC class II-related genes quantified by RT-qPCR 24 h later. Data from a representative experiment in triplicate are expressed as mean fold-change of *18s* RNA-normalized mRNA levels + SD. *E*, murine CMT167^Ctrl^ and CMT167^AhR-KO^ cells were treated with vehicle or IFNγ for 24 h and MHC class I-related genes quantified by RT-qPCR 24 h later. Data from three experiments, each in duplicate, are expressed as mean fold-change of *GAPDH*-normalized mRNA levels + SD. *F*, murine CMT167^Ctrl^ and CMT167^AhR-KO^ cells were treated with vehicle or IFNγ for 24 h and *Ciita* and *Cd74* levels quantified by RT-qPCR. Data from two experiments, each in duplicate, are expressed as fold-change of *GAPDH*-normalized means + SD. ∗*p* < 0.05, ∗∗*p* < 0.01, ∗∗∗∗*p* < 0.0001 (Student’s unpaired, two-tailed *t* test).

Some immune checkpoint and MHC-related molecules are known to be up-regulated by IFNγ in lung and other cancers through JAK/STAT signaling (51, 52, 53). Indeed, IFNγ-induction of *HLA*-*A*, *HLA-B*, *HLA-C B2M* (β2 microglobulin, a critical component of the MHC I complex) as well as *CD274* and *IDO1* in A549 cells was abrogated with Ruxolitinib, a JAK1/JAK2-specific inhibitor (54) (Fig. S1). Our RNA-seq data suggested that the AhR may contribute to expression of at least some IFNγ-inducible MHC-related molecules (*e.g.*, *HLA-A*, *B2M*, *CTIIA*, *CD74*) (Table 1). To confirm and extend these results, A549^Ctr^ and A549^AhR-KO^ cells were treated for 24 h with vehicle or 100 ng/ml IFNγ and mRNA probed by RT-qPCR for MHC class I and class II and related genes. IFNγ treatment of A549^Ctrl^ cells significantly increased expression of MHC class I molecules *HLA-A*, *HLA-B*, and *HLA-C*, and MHC class I-related *B2M* and *TAP1* (transporter associated with antigen processing) (Fig. 5*A*, first 2 bars in each graph). However, induction of these genes was significantly reduced or completely ablated in IFNγ-treated A549^AhR-KO^ cells (Fig. 5*A*, third bars). As would be expected from these results, the expression of total IFNγ-induced MHC I protein was reduced to background levels in A549^AhR-KO^ cells (Fig. 5, *B* and *C*). Furthermore, IFNγ-induced levels of MHC Class II-related CIITA (the MHC class II transactivator) and CD74 (the MHC II invariant chain) were significantly reduced in A549^AhR-KO^ cells (Fig. 5*D*). IFNγ treatment also induced high levels of MHC class I *H-2K*^*b*^, *H-2K*^*d*^, *B2m*, and *Tap1* (Fig. 5*E*) and MHC class II-related *Ciita* and *Cd74* genes (Fig. 5*F*) in murine CMT167^Ctrl^ LUAD cells. Induction of these genes was significantly reduced in CMT167^AhR-KO^ cells. Similarly, IFNγ induced *HLA-A*, *HLA-B*, *HLA-C*, *B2M, CIITA,* and *CD74*, the MHC Class II invariant chain, in human triple negative MDA-MB-231 cells (Fig. S2). As would be expected, the opposite result was seen in both the human and the murine LUAD cells following treatment with benzo(a)pyrene (B(a)P), an environmental AhR agonist found in cigarette smoke. That is, treatment with 10 μM B(a)P modestly but significantly increased human *HLA-A*, *HLA-B*, *HLA-C* and *B2M* as well as murine *H-2K*^*b*^, *H*-*2D*^*b*^, and *B2m* genes (Fig. S3). These data suggest that the AhR has the potential to influence expression of positive (MHC-related) as well as negative (IDO, PD-L1, PD-L2) immune-related molecules, specifically in tumors in which interferons are being produced.

### The AhR regulates type II interferon-induced JAK/STAT signaling and IDO1, PD-L1, and PD-L2 expression through an RNA-binding protein, HNRNPH1, and a novel lncRNA, *INCR1*

*INCR1*, an IFNγ-induced lncRNA encoded across the *CD273*/*PDCD1LG2* locus and transcribed in the anti-sense direction, was recently shown to control IFNγ induction of JAK2, STAT1, PD-L1, PD-L2, IDO1, and likely other JAK/STAT-driven immunoregulatory genes in glioblastoma, melanoma, and breast adenocarcinoma (18). *INCR1* transcripts bind to and block a ribonuclear protein, HNRNPH1, which sequesters *JAK2*, *CD274*, and *IDO1* transcripts, thereby opposing IFNγ signaling. Hence, *INCR1* upregulation and HNRNPH1 downregulation in tumors would be expected to elevate JAK2/STAT pathway and immune checkpoint genes in LUAD.

**Figure 6:**
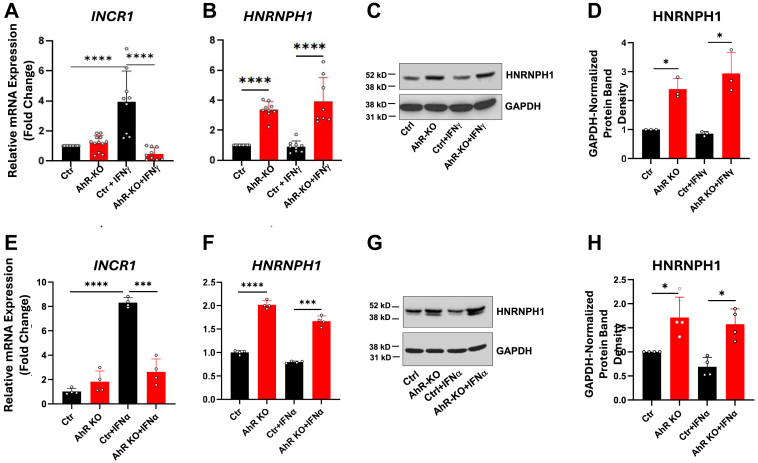
**The AhR up-regulates IFNγ- and IFNα-induced *INCR1* and represses HNRNPH1 expression.** A549^Ctrl^ and A549^AhR-KO^ cells were treated with vehicle, 100 ng/ml IFNγ, or 100 ng/ml IFNα for 24 h. *A*, RT-qPCR quantification of *INCR1* levels in four experiments, each in triplicate, are expressed as mean fold-change of *18s RNA*-normalized mRNA levels + SD. *B*, RT-qPCR quantification of *HNRNPH1* levels in four experiments, each in triplicate, is expressed as mean fold-change of *18s RNA*-normalized mRNA + SD. *C*, a representative HNRNPH1- and GAPDH-specific Western blot (n = 3). *D*, data from three western blotting experiments are presented as mean GAPDH-normalized band densities + SD. *E*–*H*, data were generated from three experiments as in “*A*–*D*” but with IFNα substituting for IFNγ. ∗*p* < 0.05, ∗∗∗*p* < 0.001, ∗∗∗∗*p* < 0.0001 (Student’s unpaired, two-tailed *t* test).

To determine if the AhR influences expression of these novel regulators of JAK/STAT signaling, the levels of *INCR1* and *HNRNPH1* were quantified by RT-qPCR in A549^Ctrl^ and A549^AhR-KO^ cells in the absence or presence of IFNγ. AhR knockout did not change the low baseline levels of *INCR1* (Fig. 6*A*, first 2 bars). As previously reported in other cancers (18), IFNγ significantly increased *INCR1* levels (third bar). Notably, the IFNγ-induced increase in *INCR1* was completely ablated in A549^AhR-KO^ cells (fourth bar), confirming a role for the AhR in *INCR1* induction. In contrast, AhR knockout significantly increased baseline levels of *HNRNPH1* mRNA (Fig. 6*B*, second bar). IFNγ did not induce baseline *HNRNPH1* levels and did not affect the increase in *HNRNPH1* after AhR knockout (Fig. 6*B*, third and fourth bars). Western immunoblotting confirmed a parallel increase in HNRNPH1 protein after AhR knockout (Fig. 6, *C* and *D*).

Similar results were obtained with IFNα-treated cells. That is, IFNα induced *INCR1* expression in control cells in an AhR-dependent fashion (Fig. 6*E*), and AhR knockout increased HNRNPH1 gene and protein expression in the absence or presence of IFNα (Fig. 6, *F*–*H*).

**Figure 7:**
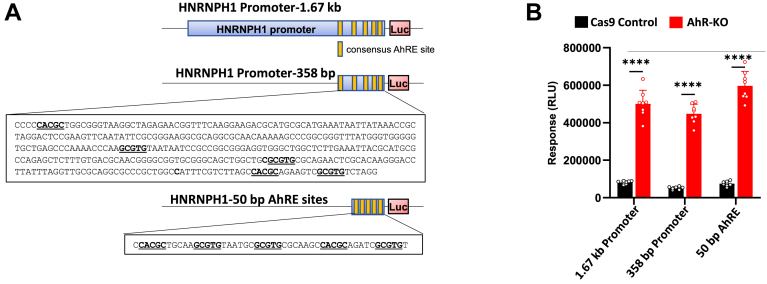
**The AhR represses transcription from the *HNRNPH1* promoter.***A*, graphic showing 1670 bp of the *HNRNPH1* promoter ligated to a luciferase coding sequence (*Top*), 358 bp of the most proximal *HNRNPH1* promoter ligated to a luciferase coding sequence with consensus AhR bindings sites (AhRE) underlined (*Middle*), and a recombinant 50 bp sequence containing all five AhR binding sites in the 1670 bp *HNRNPH1* promoter region (*Bottom*). Putative consensus AhR binding sites (5′-TNGCGTG-3) are shaded in *yellow*. The *p* value (*p* < 0.001) in this context quantifies the statistical significance of a DNA sequence matching a transcription factor's binding profile. *B*, baseline *HNRNPH1* promoter-driven luciferase reporter activity was determined in A549^Cas9^ controls and A549^AhR-KO^ cells. Data represent the means + SD from three independent experiments, n = 8 wells per condition. ∗∗∗∗*p* < 0.0001 (Student’s *t* test).

Using the Eukaryotic Promoter Database (55) and the JASPAR CORE 2018 algorithm to predict conventional AhR/ARNT binding sites (AhREs) (56), we noted that the human *HNRNPH1* promoter contains five consensus AhR binding sites (AhREs) within 358 bp of the start site (Fig. 7) (*p* < 0.001). To determine the likelihood that the AhR represses *HNRNPH1* transcription directly through at least these proximal AhREs, we constructed luciferase reporter plasmids containing 1670 bp (Fig. 7*A*, top) or 358 bp (Fig. 7*A*, middle) of the *HNRNPH1* promoter or a luciferase reporter with the five putative AhREs in a 50 kb fragment upstream of the luciferase gene (Fig. 7*A*, bottom). These reporters were transfected into A549^Cas9^ control or A549^AhR-KO^ cells, and luciferase activity was quantified 24 h later. Consistent with RT-qPCR quantification of baseline *HNRNPH1* expression after AhR-knockout, *HNRNPH1* promoter-driven luciferase activity generated with all three plasmids was significantly higher in A549^AhR-KO^ as compared with A549^Cas9^ control cells (Fig. 7*B*). These data are consistent with direct AhR-mediated repression of *HNRNPH1* transcription.

Collectively, these data demonstrate that the AhR is required for optimal IFN induction of *INCR1* and suggest that the AhR enhances JAK/STAT signaling and immune checkpoint expression through transcriptional upregulation of *INCR1* and repression of HNRNPH1.

### *INCR1* regulates JAK/STAT- and immune checkpoint-related genes in LUAD cells

**Figure 8:**
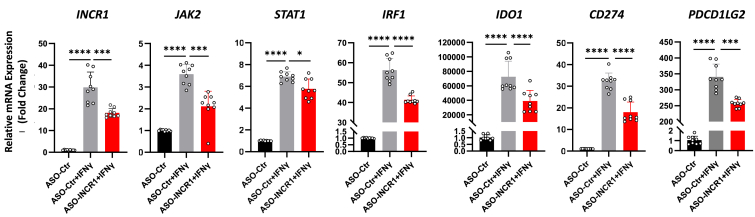
***INCR1* up-regulates IFNγ-induced *JAK2*, *STAT1*, *IRF1*, *IDO1*, *CD274*, and *PDCD1LG2*.** A549 cells were transduced with control or *INCR1*-specific anti-sense oligonucleotide (ASO), treated 24 h later with IFNγ, and assayed 24 h thereafter by RT-qPCR for expression of JAK/STAT- and immune checkpoint-related genes. Data from three experiments, each in triplicate, are presented as mean fold-change of *18s* RNA normalized mRNA + SD. ∗*p* < 0.05, ∗∗∗*p* < 0.001, ∗∗∗∗*p* < 0.0001 (Student’s unpaired, two-tailed *t* test).

Finally, we sought to confirm that *INCR1* regulates components of the IFNγ-induced JAK/STAT pathway and the resulting expression of immune checkpoints *IDO1, CD274,* and *PDCD1LG2* in human LUAD cells, as shown in other cancer types (18). *INCR1*, *JAK2*, *STAT1*, *IRF1*, *IDO1*, *CD274*, and *PDCD1LG2* were significantly induced in cells transduced with a control anti-sense oligonucleotide (ASO) and treated with IFNγ (Fig. 8, first 2 bars in all graphs). However, IFNγ-mediated induction of all of these genes was significantly reduced with transduction of an *INCR1*-specific ASO. These data are all consistent with the hypothesis that *INCR1* mediates JAK/STAT signaling and immune checkpoint expression in A549 LUAD cells and that the AhR influences that signaling by modulating *INCR1* and HNRNPH1 expression.

## Discussion

Previous studies partially mapped signaling pathways that regulate immune checkpoints critical to tumor survival. At least one such pathway is initiated by Type I and Type II interferons, immune mediators generally associated with immune enhancement but now recognized for playing a counterintuitive role by driving immunosuppression (57, 58). More specifically, interferons produced by tumor-infiltrating leukocytes induce JAK/STAT signaling within lung adenocarcinoma cells, leading to the induction of immune checkpoints PD-L1, PD-L2, and IDO1 (9, 11, 15, 48). Our interest in this pathway was piqued by a recent finding showing that the IFNγ-driven JAK/STAT pathway is regulated by two opposing factors, *INCR1* and the RNA-binding protein HNRNPH1. *INCR1* binds to HNRNPH1, preventing it from sequestering several gene transcripts including *JAK2*, *CD274*, and *IDO1* (18). A possible role for the AhR in this important pathway was suggested by our previous studies showing that the AhR regulates CD274/PD-L1 expression in oral squamous cell carcinomas (31).

**Figure 9:**
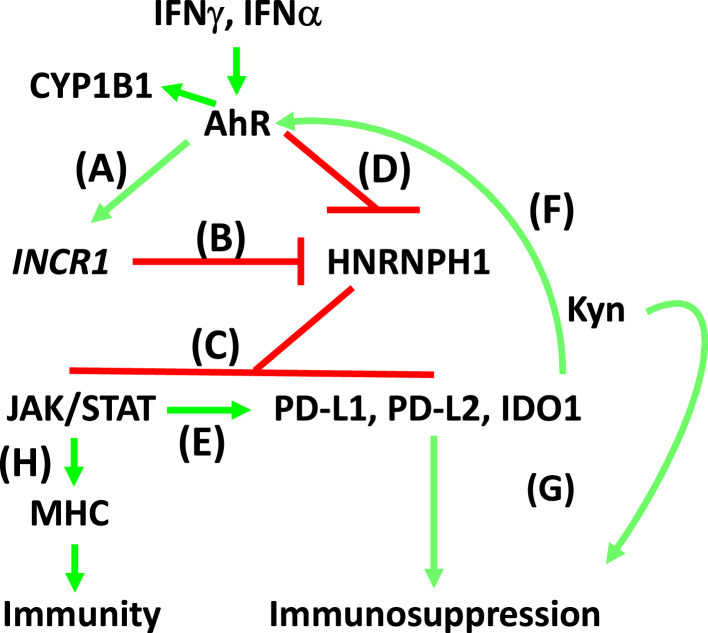
**Working model of AhR involvement in IFN signaling.***A*, the AhR upregulates *INCR1* in the presence of IFN. *B*, *INCR1* inhibits the binding of mRNAs to HNRNPH1. *C*, HNRNPH1 binds *JAK2*, *CD274*, *PDCD1LG2*, and *IDO1* transcripts thereby blocking their translation (18). *D*, the AhR also represses *HNRNPH1* transcription. *E*, the cumulative effect of the AhR on *INCR1*, HNRNPH1, and JAK/STAT pathway components is an increase in immune checkpoints PD-L1, PD-L2, and IDO1. *F*, upregulated IDO1 generates more tryptophan-derived endogenous AhR ligands in an amplification loop. *G*, PD-L1, PD-L2 and IDO-generated Kyn contribute to immunosuppression in the TME. *H*, AhR-regulated, IFN-induced JAK/STAT signaling tends to increase MHC expression which may oppose AhR-driven immunosuppression signals.

Initial RNA sequencing studies with human AhR-knockout LUAD cells suggested, and subsequent RT-qPCR experiments confirmed that the AhR is required for optimal baseline and/or IFN-induced expression of multiple JAK/STAT pathway (*STAT1*, *STAT2*, *STAT3*, *JAK2*, *IRF1,* and *IRF9*) and the immune checkpoint genes (*CD274*, *PDCD1LG2*, and *IDO1*) (Table 1). The data presented here suggest that the AhR regulates JAK/STAT and immune checkpoint levels at least in part through AhR control of *INCR1* and HNRNPH1 expression. Thus, our working model holds that the AhR alters the balance between *INCR1* and HNRNPH1 by inducing *INCR1* transcripts (Fig. 9*A*) that bind to HNRNPH1 (Fig. 9*B*) and would otherwise prevent transcription of JAK/STAT and immune checkpoint mRNAs through their sequestration or degradation (18) (Fig. 9*C*). In addition, the AhR represses *HNRNPH1* transcription (Fig. 9*D*), further compromising its ability to block JAK/STAT signaling, the end result being maximal immune checkpoint expression (Fig. 9*E*). Within this pathway, there appears to be an amplification loop consisting of AhR-mediated IDO1 expression and production of endogenous AhR ligands (Fig. 9*F*). Extending this work to a Type I IFN pathway yielded similar results (*e.g.*, Figs. 4, 6).

While the AhR interacts with the *HNRNPH1* promoter (see Fig. 7), alternative control mechanisms may be in play for the other target genes studied here, especially given that the JASPAR CORE 2018 algorithm predicts no AhR binding sites within 2000 bp of the human *INCR1*, *IRF1*, *IDO1*, *CD274*, or *PDCD1LG2* start sites, only two scattered potential AhR binding sites in the *STAT2* promoter, and only one in *IRF1*. Alternative mechanisms through which the AhR could regulate these genes may include direct promoter interaction *via* alternative AhR complexes, including AhR/KLF6 (41) or AhR/NF-κB (59, 60, 61). In addition, our studies do not rule out the likely involvement of AhR-independent contributors, including NF-κB, BRD4 (62), the PI3K/AKT/mTOR pathway, or HIF1α (63), all of which can influence PD-L1 levels.

The data summarized above point toward AhR-mediated immunosuppression in LUAD. That said, analysis of MHC class I and class II genes, as well as their associated molecules, suggests an opposing force, *i.e.*, AhR-mediated, IFN-induced expression of genes critical for immune recognition (Fig. 9*G*). In both mouse and human LUAD cells, AhR deletion reduced expression of MHC I and MHC II genes as well as genes encoding proteins critical to antigen presentation and/or MHC structure (Figs. 5, S2). This observation has important implications for recent attempts to enhance tumor immunity in patients with cancer by treating systemically with AhR inhibitors (64, 65). For example, in our hands, AhR deletion in oral squamous carcinoma cells renders mice 100% immune to further challenge with wild-type tumor cells (31). However, AhR inhibitors do not achieve the same level of protection in this (not shown) or related animal models, regardless of AhR inhibitor dose or efficacy (64, 66). This outcome could reflect a downregulation of immune checkpoints on malignant cells concomitant with a downregulation of MHC-related molecules on both the malignant cells and antigen-presenting cells within host immune compartments, possibly including tumor-infiltrating antigen-presenting cells. Thus, a relatively non-targeted systemic approach to AhR inhibition may not alter the balance between immunoenhancement (decreased immune checkpoints) and immunosuppression (decreased antigen presentation).

The central point that distinguishes this work from previous studies is the demonstration that several IFN-induced outcomes (*e.g.*, *INCR1* and HNRNPH1-regulation of the JAK/STAT pathway leading to immune checkpoint and MHC regulation) are mediated by an environmental chemical receptor, the AhR. Other specific novel findings presented in this study include the findings that: 1) the AhR regulates multiple immune checkpoint molecules in human lung adenocarcinoma cells (IDO1/IDO2, PD-L1, PD-L2), 2) the AhR is central to both type I and type II interferon signaling, 3) AhR control of IFN signaling is effected through both up-regulation of *INCR1* transcription *and* repression of *HNRNPH1* transcription, the latter likely having an effect on *INCR1* function, 4) the AhR inhibits *HNRNPH1* promoter activity suggesting a direct transcriptional regulation of *HNRNPH1* expression, and 5) the AhR controls expression of critical MHC-related molecules, including MHC class I and MHC class II molecules themselves, in human and murine LUAD cells and in human triple negative breast cancer cells. One implication of these studies is that the AhR may be involved in several other JAK/STAT-mediated cell processes. For example, the JAK/STAT pathway contributes to the regulation of apoptotic and anti-apoptotic signals (67), T cell differentiation (68), inflammation (69), lymphocyte cytokine production and cytokine receptor signaling (69), anti-viral responses (70), and angiogenesis (71). In addition, a few reports demonstrate AhR regulation of other lncRNAs (72, 73), thereby suggesting an even broader impact of the AhR on gene transcription through lncRNA modulation than previously appreciated. The involvement of the AhR in these alternative signaling pathways and the mechanisms through which it affects tumor-mediated immunosuppression remain to be determined.

## Experimental procedures

### Cell lines and treatments

The human A549 lung adenocarcinoma cell line was validated and obtained from Dr S. Mazzilli (Boston University School of Medicine), and the murine CMT167 lung adenocarcinoma cell line was validated and provided by Dr Raphael Nemenoff (University of Colorado-Denver). The A549 cell line was chosen in part because it harbors a KrasG12S mutation, a common driver mutation in human LUADs, and because it has been used extensively to study the regulation of immune checkpoints (74, 75). CMT167 cells were selected since they have commonly been used to study immune checkpoints in LUAD and since they harbor a KrasG12V mutation, another common driver mutation in human LUAD (76). Cells were cultured in Dulbecco’s Modified Eagle Medium (Corning Inc.) supplemented with 10% fetal bovine serum (Gemini Bioproducts LLC), 1% penicillin/streptomycin (Life Technologies, Gaithersburg, MD), and 1% L-glutamine (Fisher Scientific) at 37 °C with 5% carbon dioxide. Cells were kept in culture for no longer than 8 weeks, and new aliquots were thawed periodically. Cultures were confirmed to be *mycoplasma* negative every 2 months.

For *in vitro* RT-qPCR, Western immunoblotting, and immunophenotyping experiments, 50,000 or 100,000 cells/well were cultured in 12- or 6-well plates for 24 to 72 h with or without 1 to 100 ng/ml IFNγ or IFNα (PeproTech) or 10 μM benzo(a)pyrene (B(a)P) (Sigma-Aldrich).

### CRISPR/Cas9-mediated knockouts

The human AhR knockout cell line (A549^AhR-KO^) was generated using the lentiCRISPR v2 system (Addgene #52961), which includes Cas9 and a guide RNA cloning site (*BsmBI*). Two individual single-guide RNAs (sgRNAs) were designed to target the first exon of the human *AhR* gene (5′-CCTACGCCAGTCGCAAGCGG-3′ and 5′-CCGAGCGCGTCCTCATCGCG-3′) and were selected using the web resource (https://www.synthego.com/products/bioinformatics/crispr-design-tool). For the mouse AhR KO line (CMT167), two sgRNAs (5′-CGGCTTGCGCCGCTTGCGGC 3′ and 5′-AAACGTGAGTGACGGCGGGC-3′) were designed to target the first exon of the mouse *AhR* gene and were cloned into the sgOpti-vector (Addgene #85681) at the *BsmBI* site following standard procedures (77). Cas9 expression was achieved using the lentiCas9-Blast plasmid (Addgene #52962). Lentiviral particles were produced in HEK293T cells by co-transfecting the lentiCRISPRv2, sgOpti, or Cas9 expression plasmids along with lentiviral packaging plasmids (pLenti-P2A and pLenti-P2B, Cat. #LV003, Applied Biological Materials Inc.) using Lipofectamine 2000 (Invitrogen) as per the manufacturer’s instructions. Supernatants containing lentiviral particles were collected 24 and 48 h post-transfection, and cells were transduced with the lentivirus in the presence of 5 μg/ml polybrene. Forty-eight hours after transduction, A549 cells were selected with puromycin (2 μg/ml), and CMT167 cells were selected with blasticidin (5 μg/ml) and puromycin (4 μg/ml) for 2 weeks. Gene deletion was validated by DNA sequencing (Fig. S4*A*) and by Western immunoblotting (Fig. S4*B*). Control lines (A549^Cas9^, CMT167^Cas9^) were generated in an identical protocol with the Cas9 vector but without guide RNA.

### RNA extraction and bulk RNA sequencing

A549^WT^, A549^Cas9^, or A549^AhR-KO^ cells were treated for 24 h with 0.1% PBS or 100 ng/ml IFNγ, harvested, and total RNA extracted using the RNeasy Plus Mini Kit (QIAGEN) according to the manufacturer’s instructions. cDNA was generated using the High-Capacity cDNA Reverse Transcription Kit (Applied Biosystems) following the manufacturer’s instructions. There were three biological replicates for all RNA-seq studies. cDNA was sequenced using the Boston University Microarray and Sequencing Core Facility. Between 50 and 71 million total read pairs were obtained per sample. Quality metrics were similar across all samples with no technical outliers. The Broad Institute’s Morpheus software (https://software.broadinstitute.org/morpheus, Broad Institute) was used to generate heat maps. QIAGEN’s Ingenuity Pathway Analysis (IPA) software https://qiagenbioinformatics.com/products/ingenuity-pathway-analysis, QIAGEN, Inc.) was used to identify signaling pathways altered by IFNγ treatment.

### Search tool for the retrieval of interacting genes/proteins (STRING) interaction maps and gene ontology analysis of biological activity

Protein and gene interaction scores were calculated using Search tool for the retrieval of interacting genes/proteins (STRING), a precomputed resource for the collection and analysis of protein/gene associations derived from high-throughput experimental data, automated mining of public databases, and *de novo* genomic context analyses (39). The term “association” is defined as a physical binding or gene and protein participation in the same cell process(es) or metabolic pathway(s). These associations are generated from sequenced genomes that define common genomic neighborhoods, co-expression of genes, and gene fusion occurrences. Confidence scores were determined by projecting the STRING-predicted gene/protein interactions onto the Kyoto Encyclopedia of Genes and Genomes (KEGG) biological pathway database.

Common signaling pathways or cellular processes implicated by STRING analysis were identified with Gene Ontology (GO) enrichment analysis (44).

### RT-qPCR

RT-qPCR was conducted with the QuantStudio 3 Real-Time PCR System (Thermo Fisher Scientific Scientific). Relative mRNA expression was quantified using the comparative Ct (ΔΔCt) method according to the ABI manual (Applied Biosystems) and normalization to 18s RNA (human cells) or glyceraldehyde-3-phosphate dehydrogenase (*GAPDH*) (mouse cells). TaqMan assays were purchased from Thermo Fisher Scientific. The following primer pairs or TaqMan assays are listed in Table S1. In some cases, IDO1 for example, baseline levels were at the limit of detection which we considered to be a signal at 30 PCR cycles or less. Therefore, extremely large changes in fold-increases should be interpreted as a significant trend in gene up-regulation and not an exact quantification of the degree of mRNA increase.

### Western blotting

Cells were grown to 70 to 80% confluence, harvested with trypsin, and lysed with radioimmunoprecipitation assay (RIPA) buffer supplemented with protease inhibitors. Immunoblotting was performed as previously described (70). Blots were incubated overnight at 4 °C with Thermo Fisher Scientific antibodies specific for human AhR (Cat#MA1-514), HNRNPH1(Cat# PA5-50678), IDO1 (Cat#PA5-79437), or MHC-I (Cat#MA5-35712) or Cell Signaling antibodies specific for JAK2 (Cat#3230T), STAT1 (Cat#9172T), or IRF1 (Cat#8478). These specific antibodies were diluted 1:1000. GAPDH-specific antibody (Cell Signaling, Cat#97166, 1:2000) was used as loading controls. Antibodies used were validated by the manufacturers. Protein bands were visualized by the enhanced chemiluminescence (ECL) (Thermo Fisher Scientific,#32106) and iBright CL1500 Imaging System (Invitrogen). The intensity of blotting was quantified using Image J software (NIH). The relative quantification values for each protein of interest were calculated by the ratio of the target protein to the loading control within each lane and then normalized these values by dividing the ratio in each experimental lane by the ratio obtained in the control lane.

### Flow cytometry

Live cells in single cell suspensions were identified using a Fixable Live/Dead stain (BioLegend, San Diego, California) according to the manufacturer’s protocol followed by surface staining. BV421-labelled anti-PD-L1 (329,713, 1:100) and BV421-labelled anti-PD-L2 antibodies (329,615, 1:100) were purchased from BioLegend. Cell staining was quantified with a Cytek Aurora flow cytometer (Cytek Biosciences).

### *HNRNPH1* promoter deletion and luciferase reporter assays

Luciferase reporter constructs were generated using the human *HNRNPH1* promoter DNA (GeneCopoeia, Cat# HPRM76041). Three promoter fragments were created: 1) −1 to −1670 bp, 2) −1 to −358 bp, and 3) a 50 bp oligonucleotide containing five putative AhRE sites. The −1 to −1670 bp fragment was amplified *via* PCR using the following primers (5′ to 3′): forward, ACTATGGAATTCCATTCCCCTCCCCCACGCTG; reverse, TTGAAAGGATCCAGACACGCGACTTCTGCGTG. The −1 to −358 bp fragment, encompassing the five AhRE sites, was derived from the 1670 bp PCR product using restriction enzymes. The 50 bp oligonucleotide (sequence, 5′ to 3′: CCACGCTGCAAGCGTGTAATGCGCGTGCGCAAGCCACGCAGATCGCGTGT) was synthesized by Azenta (Cambridge, MA). These three fragments (1,670, 358, and 50 bp) were then cloned into the pEZX-LvPG02 luciferase reporter vector (GeneCopoeia, Cat# NEG-LvPG02) at the EcoRI/BamHI restriction sites. All constructs were verified by DNA sequencing.

For luciferase reporter assays, A549^Cas9^ control and A549^AhR-KO^ cells were seeded in 24-well plates at a density of 6 × 10^4^ cells/well in DMEM/F12 medium supplemented with 10% FBS (without antibiotics). After 24 h, cells were transiently transfected with the aforementioned reporter constructs using Lipofectamine 2000 (Thermo Fisher Scientific) following the manufacturer's protocol. Twenty-four hours post-transfection, luciferase activity was measured using the Secrete-Pair dual luminescence assay kit (GeneCopoeia, Cat# LF032) and quantified with a Synergy2 multifunction plate reader.

### *INCR1* knockdown with anti-sense oligonucleotide

A549 cells were cultured in 6-well plates and transiently transfected with 133 nM of antisense LNA GapmeR or antisense LNA GapmeR negative control (QIAGEN) using oligofectamine reagent (Invitrogen), according to the manufacturer's instructions. Twenty-four hrs after transfection, the cells were treated with IFNγ (100 ng/ml) for an additional 24 h. The *INCR1* knock-down target sequence of antisense LNA GapmeR was TTACATGATGACCTTT and antisense LNA GapmeR negative control was GCTCCCTTCAATCCAA. The knockdown efficiency of targeting *INCR1* was confirmed by RT-qPCR.

### Statistical analysis

Analysis of differentially expressed genes in the RNA-seq data sets was done with the Limma-Voom linear model analysis tool (78). The number of experiments and replicates as well as statistical tests (Student’s *t* test, ANOVA followed by Tukey’s *post hoc* test) are indicated in the figure legends. A minimum of three experiments, generally in triplicate, were performed for RT-qPCR, Western immunoblotting, and flow cytometry. Graphing and statistical analyses were performed in Prism (GraphPad). In RT-qPCR experiments, *p* values of <0.05 were considered significant. Error bars represent standard deviation of the mean (SD).

## Data availability

RNA-sequencing data will be available in GEO, December 15, 2024. All RNA-seq data will be shared on request.

## Supporting information

This article contains supporting information including Figures S1–S3 and Table S1.

## Conflict of interest

The authors declare that they have no conflicts of interest with the contents of this article.

## Supporting information

Supporting information

**Abbreviations**
AhR, Aryl hydrocarbon receptor
B(a)P, benzo(a)pyrene
B2M, Beta2 microglobulin
CIITA, MHC class II transactivator
DEG, Differentially expressed genes
GO, Gene ontology
HNRNPH1, Heterogeneous Nuclear Ribonucleoprotein H1
ICI, Immune checkpoint inhibitors
IDO1, indoleamine-2,3-dioxygenase
IFNα, Alpha interferon
IFNγ, Gamma interferon
INCR1, IFN-stimulated non-coding RNA 1
Kyn, Kynurenine
LUAD, Lung adenocarcinoma
NSCLC, Non-small cell lung cancer
PAH, Polycyclic aromatic hydrocarbons
STRING, Search tool for the retrieval of interacting genes/proteins
TAP1, Transporter associated with antigen processing
TME, Tumor microenvironment
