# Supplementary material for: The AhR regulates IFN-induced immune checkpoints in lung cancer cells through HNRNPH1, an RNA-binding protein, and INCR1, a novel long non-coding RNA
Source: J Biol Chem. 2025 May 29;301(7):110316. doi: 10.1016/j.jbc.2025.110316 (PMC12268640; doi:10.1016/j.jbc.2025.110316)
Supplement: Supporting information [file mmc1.docx]

**Supporting Information**

**Figures S1-S4, Table S1**

**The aryl hydrocarbon receptor (AhR) regulates IFN-induced immune checkpoints in lung cancer cells through HNRNPH1, an RNA-binding protein, and *INCR1*, a novel long non-coding RNA**

Brian Lara*^#^, Megan Snyder^+#^, Jocelyn Fimbres*, Eric Yang*,

Gang Song*, Vinay Kumar Duggineni*, Zhongyan Wang*^#^, and David H. Sherr*

**Supporting Figure S1**

**Figure S1. Ruxolitinib, a JAK1/2 inhibitor, blocks IFNγ-induced, JAK/STAT-mediated induction of MHC-related and immune checkpoint genes.** A549 cells were treated with vehicle (0.1% DMSO), 100 ng/ml INFγ, or IFNγ + 1 μM Ruxolitinib for 72 hours. RNA was extracted and MHC-related and immune checkpoint genes quantified by RT-qPCR. Data from four experiments are presented as the mean fold-change of 18sRNA normalized mRNA + SD. ****p<0.0001, Student’s unpaired, two-tailed T test.

**Supporting Figure S2**


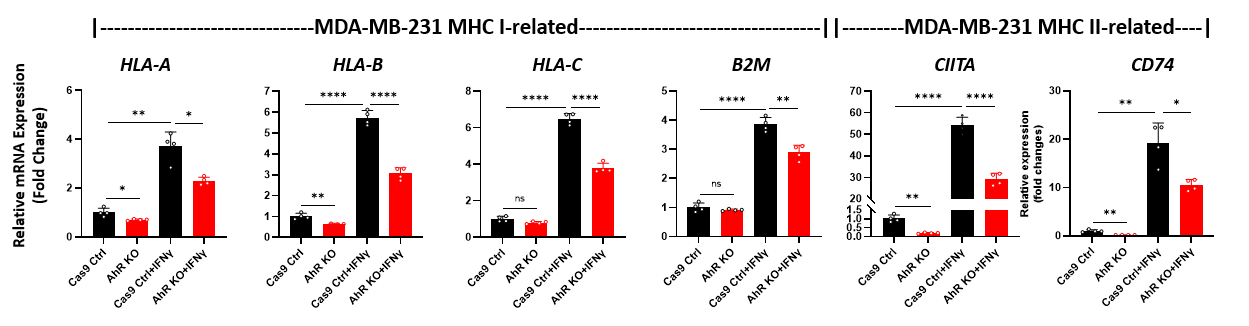


**Figure S2. The AhR regulates IFNγ-induced *MHC-I and MHC-II* expression levels in triple negative MDA-MB231 human breast cancer cells**. MDA-MB-231 Cas9^Ctrl^ or MDA-MB-231^AhR-KO^ cells were treated for 24 hrs with vehicle (0.1% PBS) or 100ng/ml IFNγ, RNA extracted, and MHC-I and II related genes quantified by RT-qPCR. Data from two experiments, each in duplicate, are presented as mean fold-change of *GAPDH* RNA-normalized mRNA levels + SD. *p<0.05, ***p<0.001, ****p<0.0001 (Student’s unpaired, two tailed T test).

**Supporting Figure S3**

**Figure S3. Benzo(a)pyrene induces MHC in human and murine LUAD cells**. Human A549 LUAD cells and murine CMT167 LUAD cells were treated for 72 hours with vehicle (0.1% DMSO) or 10 μM B(a)P and expression of MHC-related genes quantified by RT-qPCR. Data from A549 cells are *18s* RNA normalized. Data from CMT167 cells are *GAPDH* normalized. Data from four experiments, two in duplicate and three in triplicate, are presented as mean fold-change + SD. ****p<0.0001, Student’s unpaired, two-tailed T test.

**Supporting Figure S4**


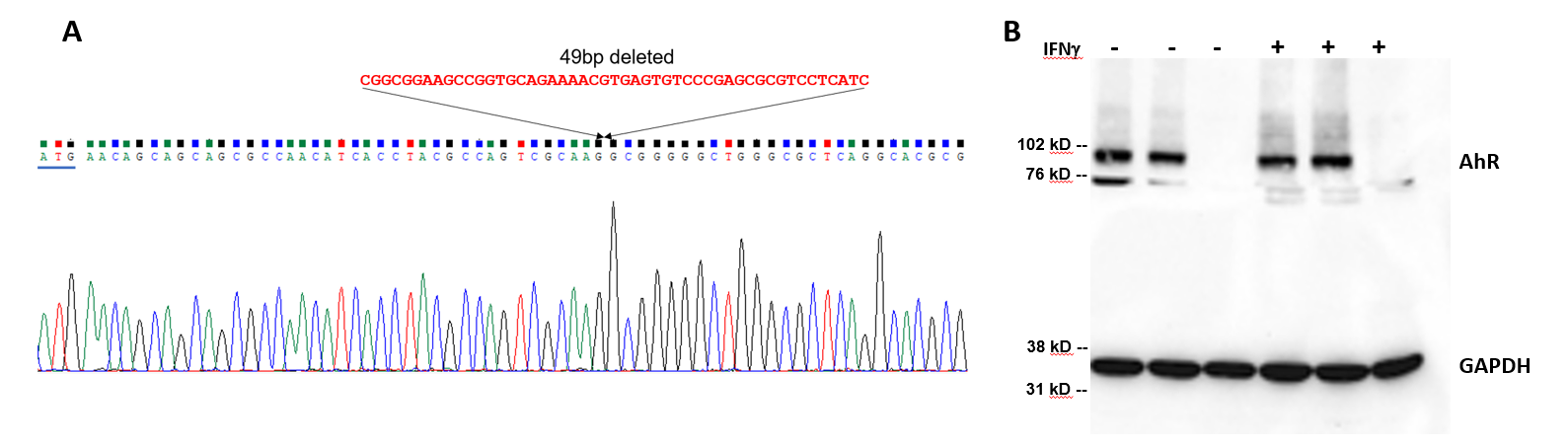


**Figure S4: Confirmation of AhR knockout. A)** Confirmation of a 49 base pair deletion in AhR exon 1 in A549^AhR-KO^ cells. **B)** A representative western blot image (n=3) confirming undetectable levels of AhR protein in A549^AhR-KO^ cells.

**Supporting Table S1**

**Table S1.** RT-qPCR primers
